# Attractive and repulsive visual aftereffects depend on stimulus contrast

**DOI:** 10.1167/jov.25.1.10

**Published:** 2025-01-09

**Authors:** Nikos Gekas, Pascal Mamassian

**Affiliations:** 1 Department of Psychology, Edinburgh Napier University, Edinburgh, UK; 2 School of Psychology, University of Nottingham, Nottingham, UK; 3 Laboratoire des Systèmes Perceptifs, Département d’études cognitives, École normale supérieure, PSL University, France

**Keywords:** adaptation, contrast, serial dependence, orientation perception, bayesian observer

## Abstract

Visual perception has been described as a dynamic process where incoming visual information is combined with what has been seen before to form the current percept. Such a process can result in multiple visual aftereffects that can be attractive toward or repulsive away from past visual stimulation. A lot of research has been conducted on what functional role the mechanisms that produce these aftereffects may play. However, there is a lack of understanding of the role of stimulus uncertainty on these aftereffects. In this study, we investigate how the contrast of a stimulus affects the serial aftereffects it induces and how the stimulus itself is affected by these effects depending on its contrast. We presented human observers with a series of Gabor patches and monitored how the perceived orientation of stimuli changed over time with the systematic manipulation of orientation and contrast of presented stimuli. We hypothesized that repulsive serial effects would be stronger for the judgment of high-contrast than low-contrast stimuli, but the other way around for attractive serial effects. Our experimental findings confirm such a strong interaction between contrast and sign of aftereffects. We present a Bayesian model observer that can explain this interaction based on two principles, the dynamic changes of orientation-tuned channels in short timescales and the slow integration of prior information over long timescales. Our findings have strong implications for our understanding of orientation perception and can inspire further work on the identification of its neural mechanisms.

## Introduction

Visual perception is a dynamic process where what is perceived now is strongly dependent on what has been perceived before. A plethora of these so-called serial effects has been identified that differ in their timescale, magnitude, and sign of correlation. In the very short-term, there is a serial dependence (SD) with the immediate past that can be attractive (Fischer & Whitney, 2014; Cichinni, Anobile, & Burr, 2014) or repulsive (Taubert, Alais, & Burr, 2016; Sun, Zhang, Alais, & Li, 2020) and has been observed in low-level (e.g., orientation: Ceylan, Herzog, & Pascucci, 2021; spatial location: Bliss, Sun, & D'Esposito, 2017) and high-level (e.g., facial features: Taubert et al., 2016; shape classification: Manassi, Kristjánsson, & Whitney, 2019) visual features. There is evidence that SD can be thought as both a perceptual and post-perceptual process (e.g., Cichinni, Mikellidou, & Burr, 2017 and Manassi, Liberman, Kosovicheva, Zhang, & Whitney, 2018 argue for the former, whereas Fritsche, Mostert, P., & de Lange, 2017 and Ceylan et al., 2021 argue for the latter). The potential functional role of SD is that the recent past can be a good predictor of the present because the environment is usually stable from moment to moment.

In spite of the importance of assuming a stable environment, there can be changes, even minute, that are crucial to an organism's survival, such as small movement of a predator in an otherwise static background. Therefore the visual system and, by extension, the organism, would benefit from the ability to increase its sensitivity to these changes. Visual adaptation is thought to play this role because it selectively decreases sensitivity to stimuli (or features) similar to the recent past but increases it for sufficiently different stimuli. This process induces aftereffects that have been documented and extensively described for many visual features (e.g., color: Webster & Mollon, 1991; orientation: He & MacLeod, 2001; motion: Schrater & Simoncelli, 1998). These aftereffects usually take the form of a negative (repulsive) correlation with the past toward the opposite end of a hypothetical perceptual continuum (e.g., towards the orthogonal orientation or the complimentary color). Although there are notable exceptions (McCollough effect: McCollough, 1965), adaptation most often operates in short to medium timescales, and the system recovers its original state after the absence of the adapting stimulus for a sufficient amount of time.

On longer timescales, many studies have shown that observers are able to learn the statistics of their environment and their perception is affected by that information consistently towards the statistics of the past (e.g., Sotiropoulous, Seitz, & Seriès, 2011; Acerbi, Wolpert, & Vijayakumar, 2012; Gekas, Chalk, Seitz, & Seriès, 2013; Chetverikov, Campana, & Kristjansson, 2017, etc.). In the even longer term, the visual system appears to develop prior expectations that are hardwired to the system and reflect the long-term statistics of the natural world (Stocker & Simoncelli, 2006; Girshick, Landy, & Simoncelli, 2011; Seriès & Seitz, 2013; De Lange, Heilbron, & Kok, 2018; Harrison, Bays, & Rideaux, 2023). The Bayesian coding framework (Mamassian, Landy, & Maloney, 2002; Knill & Pouget, 2004) has provided a successful framework for explaining these findings, where incoming information is optimally (or sub-optimally, see Rahnev & Denison, 2018) combined with prior knowledge to create the posterior percept of a stimulus.

In previous work, we looked at how all these distinct serial effects interact in orientation perception (Gekas, McDermott, & Mamassian, 2019). We showed that with systematic manipulation of stimulus orientation over different timescales, it is possible to observe and disentangle the effects of each past presentation on the current percept. With the help of a computational model, we were able to quantify the magnitude, timescale, and sign of each effect. The model assumes a continuous change in the mode of the perceptual uncertainty of the physical orientation of a stimulus (i.e., how its physical orientation shifts in the observer's perceptual space over time). A direct prediction of this model is that for a more ambiguous stimulus (and, in particular, for a stimulus of low contrast) the effects of past stimuli should decrease because the perceptual bias would be smaller. This prediction appears contrary to the Bayesian framework where more ambiguous stimuli are more affected by prior expectations. A potential solution to this contradiction may come from disentangling serial effects of different directions: repulsive aftereffects increase with higher contrast, whereas attractive aftereffects decrease with higher contrast. We argue that this can be explained by the use of two distinct mechanisms: a shift in perceptual space for repulsive aftereffects and a Bayesian integration of prior information for attractive aftereffects.

In the present study, we investigate how the contrast of a stimulus affects the serial effects it induces and how the perception of the stimulus itself is affected by these effects depending on its contrast. In an experimental paradigm based on our previous work (Gekas et al., 2019), we presented human observers with a series of Gabor patches and monitored how the perceived orientation of stimuli changed over time with the systematic manipulation of the orientation and contrast of presented stimuli. Based on the insight gained from our previous work, we hypothesized that repulsive (negative) serial effects would be stronger in the judgment of high contrast than low contrast stimuli, but the other way around for attractive (positive) serial effects. We first show experimental results that agree with our hypothesis, then we quantify the magnitude of the contrast-dependent difference in serial effects using a descriptive computational model, and, finally, we present a Bayesian model observer that provides a potential framework for the underlying mechanisms responsible for this difference.

## Experiment

### Methods

Twenty-four human participants took part in the experiment (16 females, age 25.1 ± 5.5). They all had normal or corrected-to-normal vision. All were naïve regarding the purpose of the study, and they gave informed written consent in accordance with the University of Nottingham's Ethics committee and the Declaration of Helsinki.

The experiment was completed in one session that lasted approximately one hour. At the beginning of the session, participants performed training trials on an orientation discrimination task on high (50%) and low (8%) contrast Gabor patches. For this purpose, they were asked to compare the orientation of these Gabors to a reference orientation placed within a ring probe that was placed outside the envelope of the Gabor and that remained on the screen throughout the stimulus presentation. In a two-alternative forced choice (2AFC) task, they indicated whether the orientation of the Gabor was in the orange or the blue area of the probe by pressing the corresponding key. Feedback was provided in the form of sound to familiarize participants with the task. If their performance with low contrast stimuli was worse than a specific threshold (standard deviation larger than 15°), they repeated the training with slightly increased contrast stimuli (10%). If they still performed below the threshold, the participant's data were removed from the study. No participants were removed as they all performed below the cutting threshold. Out of 24 participants, nine completed the experiment with 10% contrast stimuli, and 15 had 8% contrast stimuli.

**Figure 1.**
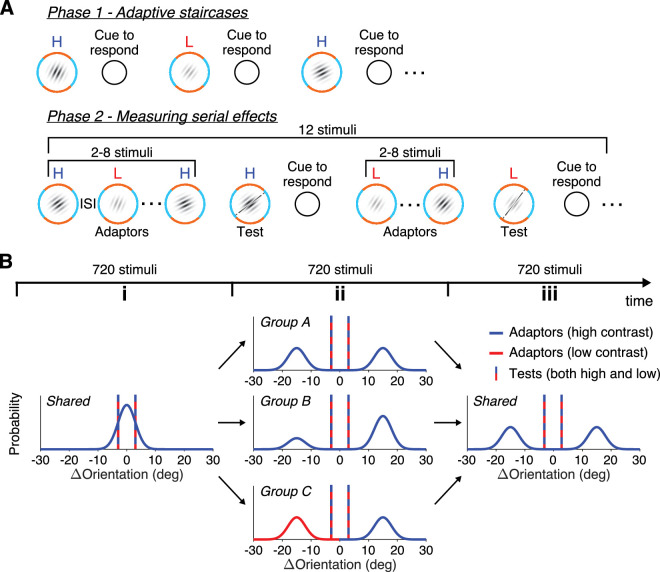
Experimental procedure and design. (**A**) The experimental session was divided into two phases. In Phase 1, participants completed a series of adaptive staircases on the perceived orientation of high and low contrast Gabors relative to a reference orientation shown by a probe ring. They were asked to indicate whether the orientation of the Gabor was in the “blue” or “orange” area of the probe. In Phase 2, participants passively observed a series of Gabors. When a cue to respond appeared, they were asked to compare the orientation of the last shown Gabor with the static probe. (**B**) Phase 2 was divided into three blocks. During the first block (i), adaptors followed a unimodal distribution around the participant's PSE for all groups. During the second block (ii), adaptor distributions differed for each group; a balanced bimodal ±15° around the PSE for Group A, an unbalanced bimodal for Group B (3:1 in favor of counterclockwise orientations), and a balanced bimodal for Group C where all clockwise orientations were shown in low contrast. During the third block (iii), adaptors followed a balanced bimodal distribution for all groups. Test stimuli (vertical lines) were shown ±3° away from the PSE at high and low contrasts across all blocks.

In the next stage of the preliminary experiment, the orientation of all stimuli was rotated by 90° clockwise so that stimuli orientations were between 0° and 90°. For convenience, we will refer to the reference as being at 45°, meaning that orientations larger than the reference (+) are in the orange area (counterclockwise of the reference), and orientations smaller than the reference (−) are in the blue area (clockwise of the reference). Participants performed eight interleaved adaptive staircases (Accelerated Stochastic Approximation, Kesten, 1958) without feedback for high and low contrast stimuli (Figure 1A, Phase 1). Two full psychometric functions, one for each contrast level, were generated from the eight staircases by fitting cumulative Gaussian functions. For each participant, the point of subjective equality (PSE) was determined as the average of the means of the two functions.

During the main part of the session (Figure 1A, Phase 2), a series of Gabors (“Adaptors”) was presented, each Gabor for a duration of 300 ms and an interstimulus interval (ISI) between Gabors of 900 ms. Participants were asked to attend to the orientation of the Gabors and wait for a circular cue to appear after a pseudo-random number of stimuli. When the cue appeared, participants were prompted to discriminate the orientation of the last presented Gabor (“Test”) by comparing it with the static reference. The next stimulus series (adaptors and test) was presented after the participant's response. The response cue appeared consistently every 12 stimuli (“Key Response”) and once more in between these 12 stimuli at random.

Participants were divided into three groups for which the presented stimuli differed: Groups “A,” “B,” and “C” (Figure 1B). As mentioned previously, stimuli consisted of “Adaptors,” to which participants did not respond, and “Tests,” to which they did respond. The orientations of the adaptors were randomly drawn from a distribution that changed twice, every third of the session (Blocks “i,” “ii,” “iii”). In Block “i,” the orientation of adaptors was drawn from a Gaussian distribution centered at each participant's PSE and with a standard deviation of 3° for all participants (of all three groups). In Block “iii,” again for all participants, the distribution of adaptors was changed to a bimodal distribution with modes at ±15° from the PSE and standard deviation of 3°. In Block “ii,” the distribution of adaptors was different for each group. For Group A, the distribution was bimodal and balanced across the two modes, just like the last block. For Group B, the distribution of adaptors was bimodal but imbalanced, such that the mode centered at 15° was three times more likely than the mode at −15°. Finally, for Group C, the distribution was balanced bimodal, but all adaptors clockwise of the PSE (negative values) were low-contrast stimuli. All other adaptors across Groups and Blocks were presented with high contrast.

The adaptor distributions of each group play distinct roles in the experimental design. Group A serves as a baseline for comparing Groups B and C. During Blocks ii and iii, the bimodal distribution is balanced, so participants’ PSEs are not expected to be biased on average. However, their sensitivity is expected to decrease due to the repulsive effects of the adaptors leading to more varied responses. For Group B, we expect that participants’ perceptions will be biased away from the dominant orientation (15^o^) during Block ii, resulting in PSEs shifting toward positive values, whereas in Block iii, we expect PSEs to move slightly toward negative because of a weak attractive effect. In Group C, a similar pattern is expected because of the contrast difference between the clockwise and counterclockwise adaptors. Despite being equally likely to be presented, high-contrast adaptors are expected to induce a repulsive bias during Block ii and an attractive bias during Block iii, if the contrast of the adaptor influences the magnitude of serial effects.

The orientations of the test Gabors were ±3° away from each participant's PSE and could be either high (HC) or low contrast (LC), thus creating four types of tests: HC+, LC+, HC−, and LC−. Participants were presented with 2160 Gabor stimuli in total and were asked to respond 360 times. Every 240 trials, participants were asked to respond 40 times (i.e., 10 times of each type of test stimulus in a randomized order).

All stimuli were generated using the MATLAB programming language with the psychophysics toolbox (Brainard, 1997; Pelli, 1997) and displayed on a calibrated LCD monitor with a resolution of 1920 × 1080 pixels at 60 Hz. Participants viewed the display in a darkened room at a viewing distance of 60 cm, and a chin rest was used to maintain a constant head location and viewing distance.

### Results

We investigated how the contrast of the stimulus to be judged is modulating the effects of past stimuli presentations. Participants viewed serially presented Gabors and, upon the appearance of a cue, were asked to compare the orientation of the last shown Gabor with that of a static reference. Three different groups of participants were tested. For all groups, in the first and last third of the session (blocks i and iii), the adaptors (i.e., the stimuli that participants did not respond to) were drawn from the same distributions. However, the middle third of the session (block ii) differed for each group. For Group A, the unimodal distribution of adaptors was changed to a balanced bimodal distribution. For Group B, the bimodal distribution was unbalanced so that counterclockwise orientations were three times more likely to appear than clockwise orientations. For Group C, the bimodal distribution was balanced, as in Group A, but all clockwise stimuli were shown with low contrast.

**Figure 2.**
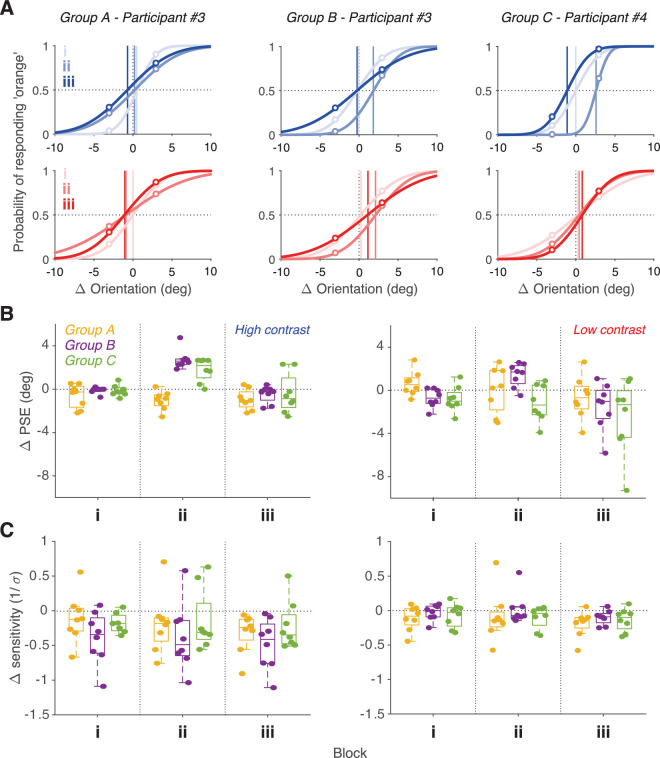
Experimental results. (**A**) Psychometric functions of indicative participants from each group are shown for each experimental block for high (blue curves) and low (red curves) contrast stimuli. Vertical colored lines indicate the PSE for each block. (**B**) Averaged shifts in the PSE in comparison to the one measured in Phase 1 are shown for each block and group for high (left) and low (right) contrast test stimuli. Box plots show the median, the interquartile range and the minimum and maximum values. Colored dots show individual data. (**C**) Averaged shifts in the sensitivity in comparison to the one measured in Phase 1 are shown for each block and group for high (left) and low (right) contrast test stimuli.

Because two orientations are sampled clockwise and counterclockwise of the reference, psychometric functions can be generated for each participant by fitting their responses to a normal cumulative distribution. Psychometric functions for three example participants, one from each group, are plotted for each contrast and block in Figure 2A. Responses and functions are shown in relation to the original PSE measured during Phase 1. For the participant in Group A, the psychometric functions of high-contrast test stimuli (top plot) are sharper than those of low contrast (bottom plot), as expected. The functions of Block i are steeper than those of Blocks ii and iii, for both contrast levels, suggesting that the test stimuli became less reliable with the second Block. The PSE (shown by the vertical line in the plots) varied little across Blocks, indicating that the change of sensitivity was not accompanied by a bias of the distribution of adaptor orientations.

For the participant of Group B, there is a clear shift of the psychometric function during Block ii, as expected from the overexposure to counterclockwise adaptors. Responses to stimuli clockwise of the reference are more consistent, whereas responses to stimuli counterclockwise of the reference are less consistent. The PSE has shifted counterclockwise, but the slope of the function has remained similar to that of Block i. During Block iii, the PSEs return to their original orientations, whereas the slopes of the functions are flatter than in the previous blocks, similarly to the participant of Group A.

For the participant of Group C, there is a clear distinction between the effect of adaptor manipulation to high- and low-contrast test stimuli. In Block ii, there is a strong counterclockwise shift of the PSE but only for high contrast test stimuli. There is no shift for low contrast test stimuli. As in the other two groups, in Block iii, PSEs return to their original orientations, and there is a decrease in the steepness of the functions.

These individual trends are representative of the behavior of the groups. Figure 2B plots the evolution of each group of participants’ averaged shifts in the PSE over time. Looking at Block i, which is identical for each group, on average, there is no or minimal shift to the PSE as measured from the adaptive staircases. PSE shifts in Blocks ii and iii show the overall effects of each group manipulation. For Group A, there is no significant change to the PSEs irrespective of stimulus contrast. For Group B, PSEs shift counterclockwise in Block ii, more so for high-contrast stimuli, then return to their original values in Block iii. For Group C, in Block ii, high-contrast tests are more likely to be perceived as clockwise than before, as in Group B. However, this effect is not present for low-contrast tests. PSEs in the last block return to their original values. A mixed-model analysis of variance was used to examine the significance of the main effects and interactions of Group (between subjects), and Contrast and Block (within subjects). Greenhouse-Geisser corrections were applied when data violated the assumption of sphericity. There was no significant effect for Group (*F*(2, 21) = 1.46, *p* = 0.254, $\eta_{p}^{2}=0.122$) but there was a significant effect for Contrast (*F*(1, 42) = 5.57, *p* = 0.028, $\eta_{p}^{2}=0.21$) and Block (*F*(1.52, 31.9) = 22.72, *p* < 0.001, $\eta_{p}^{2}=0.52$), and significant two-way interactions between Group and Block (*F*(3.04, 31.9) = 7.13, *p* < 0.001, $\eta_{p}^{2}=0.405$), between Group and Contrast (*F*(2, 21) = 8.16, *p* = 0.002, $\eta_{p}^{2}=0.437$), and between Block and Contrast (*F*(1.41, 29.55) = 3.72, *p* = 0.05, $\eta_{p}^{2}=0.15$).

Looking at changes in sensitivity (Figure 2C), there are no clear trends except for the decrease in sensitivity at high contrast in comparison to the adaptive staircases. This could relate to the attentional demands of Phase 2 and possible visual fatigue to the participants. Sensitivity appears to slightly decrease in Block iii across all groups as predicted; however, a mixed-model analysis of variance revealed no significant main effects or interactions between Group, Contrast, and Block.

The results overall suggest that manipulation of stimulus contrast has two clear consequences to serial effects. First, repulsive aftereffects are weaker in lower-contrast test stimuli. Second, lower-contrast adaptor stimuli produce weaker repulsive aftereffects. We hypothesized that a difference should also be present for attractive aftereffects, but these are significantly weaker than repulsive aftereffects and it is difficult to evaluate them just by looking at the shifts in PSEs or sensitivity. A computational model (“Linear model”) is therefore used to quantify the magnitude and timescales of different serial effects and determine how they differ for high- and low-contrast stimuli.

### Linear model

#### Description

The Linear model assumes that the perceived orientation of the current stimulus is affected by the perceived orientations of past stimuli according to a function *F*. It is fully described in Gekas et al. (2019), but we also present a condensed description here (Supplementary Figure S1) along with the changes that allow the model to take into account the contrast of the stimulus.

The probability of responding counterclockwise of the 45° reference (i.e., “orange”) *p_s_* for a test stimulus *s* with physical orientation θ_*s*_ is the value of the psychometric function with midpoint *c_s_* at θ_*s*_ (Supplementary Figure S1A). The psychometric function is a logistic function defined as

(1) $$\begin{array}{c} p_{s}=\frac{1}{1+e^{-k\left(\theta_{s}-c_{s}\right)}}\; \end{array}$$

where *k* is the slope at the midpoint. For different contrast levels, the midpoint slope differs, i.e., *k* = *k_high_* for high-contrast stimuli and *k* = *k_low_* for low-contrast. The model assumes that the history of past stimuli shifts horizontally the whole psychometric function, so that the original midpoint *c*_0_ moves to a new midpoint:

(2) $$\begin{array}{c} c_{s}=c_{0}+shift\; \end{array}$$

The *shift* is calculated by taking the orientation difference between each past stimulus $\theta_{s-1}...\theta_{s-n}$ and the midpoint of the function at that time $c_{s-1}...c_{s-n}$, and multiplying this difference with a function *F* that captures the influence of each past stimulus on the shift

(3) $$\begin{array}{c} shift_{stimuli}=k_{norm}\sum_{i=1}^{S}\left(c_{s-i}-\theta_{s-i}\right)\cdot F\left(i\right)\; \end{array}$$

where *S* is the number of all stimuli presented before stimulus *s*, and *k_norm_* is normalizing the magnitude of the function across participants and is defined as *k_norm_* = 2 · ln (9)/*k*. Without the influence of past stimuli, the shift regresses to zero.

The function *F* has as many degrees of freedom as there are past stimuli. However, we assume that this function is continuous and relatively smooth, and to a first approximation, we assume that it is a linear summation of simple linear functions

(4) $$\begin{array}{c} F=\;f_{1}+f_{2}+f_{3}+...\; \end{array}$$

Each linear function $f_{1},\;f_{2},\;...$ is defined by two parameters: (1) the initial weight *w* of the one-back stimulus, and (2) the number of stimuli *m* it takes for that weight to reach zero (Supplementary Figure S1B). The value of the function for each preceding stimulus *i* is calculated as follows:

(5) $$\begin{array}{c} f\left(i\right)=\left\{\begin{array}{c} \gamma \left(w+\frac{-w\cdot \left(i-1\right)}{m}\right),\;\mathrm{when}\;1\leq i\leq m\\ 0,\;\mathrm{when}\;i>m \end{array}\right.\; \end{array}$$

where γ indicates the relative strength between a high contrast and a low contrast adaptor,

(6) $$\begin{array}{c} \gamma =\frac{w_{high\_contrast}}{w_{low\_contrast}}\; \end{array}$$

The complexity of function *F* can increase by adding more linear components and summing them. The resulting function is a piecewise linear function with an even and a moderate number of free parameters.

To fit the model's parameters to the data, we calculate the log likelihood of obtaining each participant's responses

(7) $$\begin{array}{c} \;\log L\left(p\right)=\sum_{i=1}^{R}r_{i}\cdot \log\left(p_{i}\right)+\sum_{i=1}^{R}\left(1-r_{i}\right)\cdot \log\left(1-p_{i}\right) \end{array}$$

where *R* is the number of all responses given by the participant during the experiment, and responses *r* are normalized between 0 for “blue” and 1 for “orange.” The model's parameters are the ones that maximize the log likelihood. For each participant, the parameters *c*_0_, *k_high_*, and *k_low_* (Equation 1) are calculated from the staircases of Phase 1. The parameters for function *F* (Equations 4 and 5) are free parameters that are fitted to responses across all participants from all groups, a total of 24 × 360 = 8640 data points.

### Results

**Figure 3.**
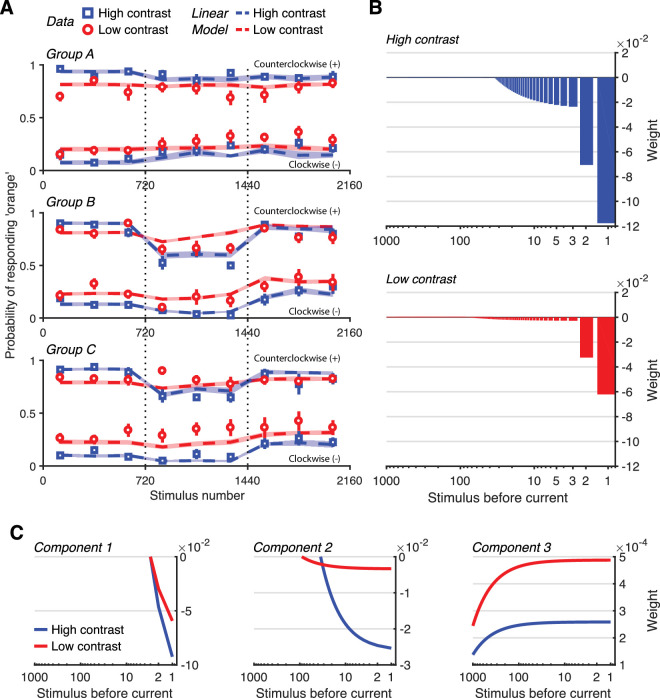
Linear model fits and components. (**A**) The averaged probability of responding “orange” to test stimuli is plotted for each group for high- (blue squares) and low- (red circles) contrast tests. Error bars indicate SEM. Colored dashed lines indicate the fit of the linear model to the experimental data. The area around the line indicates SEM. (**B**) The weight of each past stimulus is shown against the stimulus's position in time, according to the linear model. Blue bars (top) show the weights to high contrast tests, whereas red bars (bottom) show the weights to low contrast. (**C**) Each of the components of the linear model are shown separately for high- (blue lines) and low- (red lines) contrast tests. Components differ in their sign (positive and negative) and timescale (very short to very long). Note the different scales on the y-axis for the three plots.

Figure 3A plots the evolution of participant's responses over time separately for each Group. These are the same data that are used to produce the psychometric functions in Figure 2. Blue squares indicate the average probability of responding counter-clockwise (“orange”) over 10 responses (across 240 trials) for high-contrast test Gabors, whereas red circles indicate the same for low contrast tests. Looking at Block i, which is identical for all groups, tests that are counter-clockwise of the reference (HC+ and LC+) are on average more likely to induce a counterclockwise response (toward 1), whereas the opposite is true for counterclockwise tests (HC− and LC−, towards 0). Also, counterclockwise high-contrast tests (HC+) are more likely to induce a counterclockwise response than low-contrast counterclockwise tests (LC+) and likewise for clockwise high-contrast tests (HC−) and clockwise low contrast tests (LC−).

The responses during Blocks ii and iii again show the overall effects of adaptor manipulation. As an example, for participants of Group B, responses in Block ii to counterclockwise tests (HC+ and LC+) are strongly affected by the distribution of adaptors and that effect is stronger for high-contrast than low-contrast tests, because average responses to high-contrast tests are close to chance level (0.5) and more variable than responses to the corresponding low-contrast tests. In Block iii, when the adaptor distribution becomes balanced, this bias is eliminated.

The fits of the linear model for each type of test stimulus are shown as dashed lines in Figure 3A. The exact parameters of the Linear model can be seen in Supplementary Table S1. The model can capture the biases in the different groups for high and low contrast stimuli. For example, the model correctly predicts very small change in the responses to low contrast stimuli in Group C while still predicting the shift for high contrast stimuli. Figure 3B shows the weight of each past stimulus presentation to high- (top) and low- (bottom) contrast tests. In both cases, the immediately preceding stimulus has a strong negative (repulsive) effect to the perceived orientation of the current stimulus. Stimuli further in the past have a similar negative but increasingly diminished effect until approximately 50 stimuli in the past where the effect reverses sign and becomes positive (attractive) but significantly weaker. Although the pattern is similar for high and low contrasts, the magnitude of the effects differs; negative effects are stronger for high-contrast than low-contrast tests, whereas positive effects are weaker.

The discrepancy between the influence functions for high- and low-contrast tests is easier to see if the components that make up the piece-wise linear functions are plotted separately. Figure 3C plots the three components for the two contrasts. The left panel shows the first component that reveals a strong negative dependence on the two stimuli that precede the test Gabor. The time scale is the same, but the magnitude is halved for low-contrast stimuli. The middle panel shows the second component that reveals a weaker negative dependence but over a longer timescale. The effect appears to last longer for low-contrast stimuli, but the magnitude is many times smaller. The right panel shows the third component that reveals the long-term positive influence of stimuli. In this case, the effect is stronger for low-contrast stimuli and appears to last longer as well. It is important to note that the third positive component appears to start from the immediately preceding stimulus, but this small positive weight is swamped by the much stronger negative weights of the other two components. Finally, the value of the γ parameter fitted by the model was 2.56, suggesting that the weights, and thus influence, of high contrast adaptor stimuli was several times stronger than the influence of low-contrast stimuli.

The Linear model provides a good description of the sign, magnitude, and timescales of serial effects but it does not provide a possible explanation of how these serial effects are produced and why they behave differently for stimuli of different contrast. We now present a Bayesian model that provides one possible framework for the neural mechanisms that produce these effects and can explain why contrast discrepancies arise.

### Bayesian model

#### Description

The main insight from the experimental results and the Linear model is that repulsive aftereffects are stronger at high contrast while attractive aftereffects are stronger at low contrast. Repulsive aftereffects are strongly associated with adaptation which can be modeled with changes in the tuning curves of orientation-tuned neurons (or channels) (e.g., a decrease in gain, a decrease in sensitivity, or a shift in the preferred orientation of the neuron [Dragoi, Sharma, & Sur, 2000; Kohn, 2007]). Attractive aftereffects are strongly associated with the Bayesian coding hypothesis of perception according to which the likelihood of the perceived feature of a stimulus is combined with a prior expectation to produce a posterior probability of the stimulus feature.

**Figure 4.**
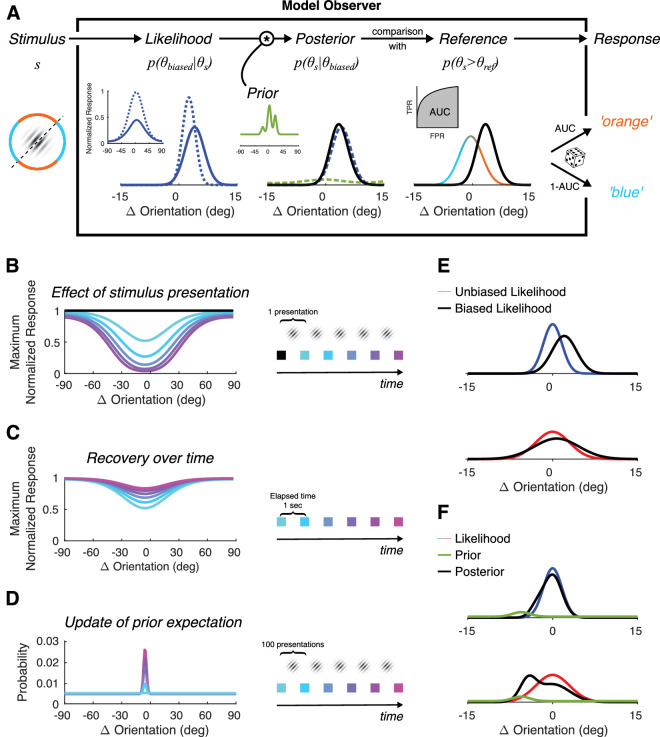
Bayesian model description. All effects correspond to the parameters of the best-fitting V5-CV model. (**A**) An overview of the model observer. A stimulus’ orientation is encoded by a network of orientation-tuned channels. The likelihood is combined with a prior expectation to produce a posterior distribution. The posterior is compared with the distribution of the reference and a response is generated. (**B**) The effect that a stimulus presentation has on the network of orientation-tuned channels. Each stimulus presentation reduces the maximum response of the network based on its orientation, contrast, and duration. The colored lines show the decrease in response for successive stimuli presentation in the absence of recovery. (**C**) The recovery rate of the network over time. The maximum response of the network slowly regresses to its initial value over time. The colored lines show the recovery every one second. (**D**) The rate of developing prior expectations. Each stimulus presentation affects the prior expectation over orientation. The rate is very slow so hundreds of presentations are required for the prior to change as shown by the colored lines. (**E**) Example of likelihood shifts for high and low contrast stimuli. The change in the network gain has a bigger effect on high than low contrast stimuli. (**F**) Example of prior influence for high and low contrast stimuli. The prior has a larger effect on low than high contrast stimuli.

We implemented a Bayesian observer that combines these two properties and that can explain the interaction between contrast and sign of the aftereffects. Figure 4A provides an overview of the model observer. First, the orientation likelihood of a stimulus is encoded by a bank of orientation-tuned channels. Next, the likelihood is combined with a prior expectation of orientations to produce a posterior distribution. Finally, the probability of the observer responding “blue” or “orange” is derived from the comparison of the posterior with the orientation distribution of the reference.

More specifically, orientation is encoded in a bank of orientation-tuned channels φ (*N* = 60, equally spread) defined as von Mises (circular normal) distributions

(8) $$\begin{array}{c} \varphi_{i}\left(\theta \right)∼V\left(\mu_{i},\kappa_{\varphi }\right),\; \end{array}$$

where µ_*i*_ is the preferred orientation of channel φ_*i*_ and κ_φ_ is the channel's spread. Each channel produces a response *n* to a stimulus *s* depending on the orientation θ_*s*_ and contrast *c_s_* of the stimulus. When contrast is maximum (i.e., *c_s_* = 1), the response of each channel *n_i_* is computed from a von Mises distribution

(9) $$\begin{array}{c} n_{i}\left(\theta_{s},c_{s}=1\right)=V\left(\theta_{s}|\mu_{i},\kappa_{\varphi }\right),\; \end{array}$$

where θ_*s*_ is the orientation of the stimulus and κ_φ_ is the spread at maximum contrast. The response distribution is normalized to [0, 1], where 1 indicates maximum response of a channel and 0 no response at all. As contrast decreases, the response of a channel is calculated from

(10) $$\begin{array}{c} n_{i}\left(\theta_{s},c_{s}\right)=\frac{c_{s}^{c_{exp}}}{c_{50}^{c_{exp}}+c_{s}^{c_{exp}}}V\left(\theta_{s}|\mu_{i},\kappa_{c}\right),\; \end{array}$$

where *c*_50_ is the semi-saturation constant, *c_exp_* is the exponent, and κ_*c*_ is the spread at contrast *c*. While it is usually assumed that orientation bandwidth is contrast invariant, it has been shown to decrease with contrast especially for stimuli presented at variable contrasts (Nowak & Barone, 2009). We used a saturation function for the decrease in tuning width so that κ_*c*_ is calculated from

(11) $$\begin{array}{c} \kappa_{c}=\frac{c_{s}^{ɛ}}{w_{50}^{ɛ}+c_{s}^{ɛ}}\kappa_{\varphi },\; \end{array}$$

where *w*_50_ is the semi-saturation constant, ε is the exponent, and κ_φ_ is the spread at maximum contrast.

The response of each channel is multiplied by the logarithm of its tuning curve log φ_*i*_ (at maximum contrast), and the overall log likelihood log *L_s_* of the stimulus’ orientation is computed from the sum of all channels. In the absence of any serial effects, the orientation likelihood *p*(θ_*observed*_|θ_*s*_) is

(12) $$\begin{array}{c} p\left(\theta_{observed}|\theta_{s}\right)=\exp\left(\sum_{i}\omega_{s}n_{i}\left(\theta_{s},c_{s}\right)\;\log\varphi_{i}\right),\; \end{array}$$

where ω_*s*_ is an accumulation parameter corresponding to the duration of the stimulus, so that the more a stimulus is presented, the sharper the likelihood becomes. Figure 4A shows an example of the response of the network to a high-contrast stimulus (inset, dashed blue curve) and the corresponding orientation likelihood (main figure, dashed blue curve) in the absence of any gain reduction in the network.

A stimulus presentation at trial *t* decreases the maximum possible response $m_{i}^{t}$ of each channel by

(13) $$\begin{array}{c} m_{i}^{t}=m_{i}^{t-1}\left(1-\alpha \;n_{i}\left(\theta_{s},c_{s}\right)\right),\; \end{array}$$

where α specifies the relation between the activation of a channel by the stimulus *s* at time (*t* − 1) and the decrease in response by the channel, and is assumed to be constant across channels. So, a high-contrast stimulus will produce a larger decrease than a low-contrast stimulus. Because the decrease depends on the maximum possible response of the channel at time (*t* − 1), the effect is self-constrained for repeated stimulus presentations. Figure 4B illustrates an example of the decrease in maximum normalized response across the bank of channels after five sequential presentations of the same stimulus with θ_*s*_ = −5.66°, *c_s_* = 0.5 and duration 300 ms (assuming without any recovery that we define next).

A channel's response is normalized to [0, 1] so the initial maximum response of a channel is assumed to be 1 (i.e., $m_{i}^{t=0}=1$). In the absence of stimuli, the maximum response regresses to its initial value by

(14) $$\begin{array}{c} \;m_{i}^{t}=m_{i}^{t-1}\left(1-\min\left[\beta \;\Delta t,1\right]\right)+\min\left[\beta \;\Delta t,1\right],\; \end{array}$$

where β is a recovery constant multiplied with time elapsed between trials Δ*t* (in seconds) capped at 1 (full recovery). The time between trials is extracted directly from the data, so it can vary in some cases (e.g., after participant responses or during breaks). Figure 4C illustrates an example of the recovery of maximum response after one stimulus presentation in the absence of stimuli for five time-steps of one second.

In the example of Figure 4A, the past stimuli presentations have altered the response profile of the bank of channels, so that the response to the same stimulus is reduced and biased (inset figure, solid blue curve). Because of the change in the state of the channels’ population, the likelihood to stimulus *s* is now

(15) $$\begin{array}{c} \;p\left(\theta_{observed}|\theta_{s}\right)=\exp\left(\sum_{i}\omega_{s}m_{i}n_{i}\left(\theta_{s},c_{s}\right)\;\log\varphi_{i}\right),\; \end{array}$$

which is shifted away from the original (Figure 4A, solid blue curve). Although it may seem counterintuitive, the change of the bank of channels’ response has a larger effect on high contrast stimuli than on low contrast ones. Figure 4E illustrates that difference. Blue and red curves represent the likelihood of a high and low stimulus, respectively, with orientation θ_*s*_ = 0° for an unaffected bank of channels. The black curves indicate the likelihoods after one stimulus presentation with θ_*s*_ = −5.66° and *c_s_* = 0.5. This difference in likelihood shift corresponds to the difference in the weights of components 1 and 2 of the Linear model.

The likelihood is combined with the prior expectation *p*(θ_*s*_) over all orientations to generate the posterior distribution

(16) $$\begin{array}{c} p\left(\theta_{s}|\theta_{observed}\right)\propto p\left(\theta_{observed}|\theta_{s}\right)\cdot p\left(\theta_{s}\right).\; \end{array}$$

The prior is updated with each stimulus presentation, so that the prior distribution at trial *t* is

(17) $$\begin{array}{ccc} & & \;p^{t}\left(\theta_{s}\right)=\left(1\;-\;\tau \right)p^{t-1}\left(\theta_{s}\right)+\tau \cdot p^{t-1}\left(\theta_{s}|\theta_{observed}\right),\; \end{array}$$

where τ is the rate of integration of the current estimation to update the prior. Figure 4D illustrates an example of the evolution of the prior after multiple presentations of the same stimulus. Because the rate is very low, the prior changes substantially only after hundreds of stimuli presentations. In our example of Figure 4A, the prior at the beginning of block iii for a participant of Group B is shown in the inset figure. In contrast to the shift of the likelihood, the prior has a larger effect on low than high contrast stimuli, because a low contrast stimulus is more unreliable and, therefore, its likelihood distribution is wider than the high contrast one. Figure 4F illustrates this property using the prior from Figure 4D and the same likelihoods from 4E. The difference of prior influence corresponds to component 3 of the Linear model.

The perception of the reference is itself unreliable and varies greatly between subjects, which is reflected in their initial PSEs. The probability of the reference is described in the model by a von Mises distribution so that *p*(θ_*ref*_) = *V*(μ_*ref*_,κ_*ref*_), where µ_*ref*_ and κ_*ref*_ represent the mean and spread of the distribution respectively. The probability of the stimulus orientation being more clockwise than the reference orientation (i.e., in the “orange” area) *p*(θ_*s*_ > θ_*ref*_) is calculated from the Area Under the Receiver Operating Characteristics (ROC) Curve (AUC) between the posterior and the reference distributions

(18) $$\begin{array}{c} p\left(\theta_{s}>\theta_{ref}\right)=AUC\left(p\left(\theta_{s}|\theta_{observed}\right),p\left(\theta_{ref}\right)\right).\; \end{array}$$

As with the Linear model, we choose the parameters that maximize the log likelihood of obtaining each participant's responses (see Equation 7)

(19) $$\begin{array}{ccc} & & \;\log L\left(p\right)=\sum_{i=1}^{R}r_{i}\cdot \log\left(p_{i}\right)+\sum_{i=1}^{R}\left(1-r_{i}\right)\cdot \log\left(1-p_{i}\right),\; \end{array}$$

where *R* is the number of all responses given by the participant during the experiment, and responses *r* are normalized to 0 for “blue” and 1 for “orange.”

A summary of the parameters used in the model can be seen in Supplementary Table S2. The parameters for the channel's maximum tuning width κ_φ_ and the response contrast saturation *c*_50_ and *c_exp_* were taken from Vanni, Hokkanen, Werner, and Angelucci (2020). In their meta-study, they reported properties of visual areas V1, V2, and V5/MT in the primate genus Macaca. For each of our participants, we use the staircases of Phase 1 to define parameters µ_*ref*_ and κ_*ref*_. The parameters for the serial effects α, β, and τ, the response accumulation parameter ω and the tuning width saturation parameters *w*_50_ and ε were free parameters fitted to responses across all participants from all groups, a total of 24 × 360 = 8640 data points.

### Results

**Figure 5.**
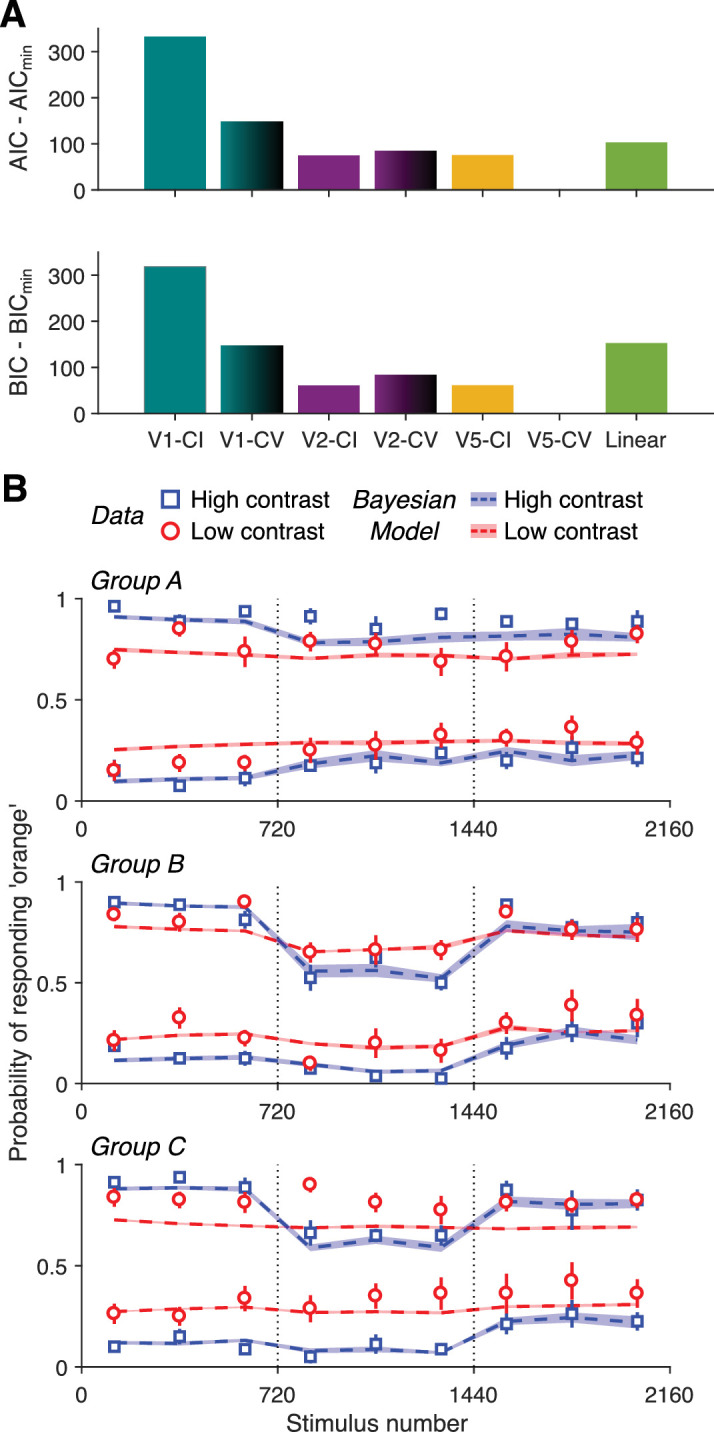
Bayesian model comparison and model fits. (**A**) Model comparison. The differences between the Akaike information criterion (top) and Bayesian information criterion (bottom) values of all Bayesian models and the Linear model with the value of the best-fitting model (V5-CV) is shown. (**B**) Model fits. The averaged probability of responding “orange” to test stimuli is plotted for each group for high (blue squares) and low (red circles) contrast tests. Error bars indicate SEM. Colored dashed lines indicate the fit of the best fitting Bayesian model (V5-CV) to the experimental data. The areas around the lines indicate SEM.

Because there is no consensus on the properties of orientation channels in human observers, we used the parameters reported by Vanni et al. (2020) for neurons at different visual areas, specifically V1, V2, and V5/MT. We used median values for orientation bandwidth, contrast semi-saturation and exponent. We also implemented two versions of each model, one in which there was contrast invariance (CI) on channel tuning width, and one in which there was contrast variance (CV). So, there were a total of six different Bayesian models. Figure 5A shows a comparison between the Akaike information criterion and Bayesian information criterion values of each Bayesian model plus the linear model. For both metrics, the model V5-CV performs better than any other model (i.e., the model that uses parameters found in neurons of areas V5/MT and assumes contrast variance for the tuning width). In addition, a likelihood ratio test between V5-CI (as the restricted model) and V5-CV (as the unrestricted model) suggests that the unrestricted model is significantly better than the restricted one (*p* < 0.001). It is important to note here that the Bayesian models and the linear model do not differ significantly in their ability to explain the variance in the data. The best performing V5-CV model can explain 39.05% of the variance, whereas the linear model can explain 38.19% (adjusted *R*^2^). However, even the worst performing V1-CI model can explain 37.08% of the variance. A comparison of the fits of the Bayesian model (V5-CV) and the linear model regarding the PSE and sensitivity shifts (as plotted in Figures 2B and 2C) can be seen in Supplementary Figure S4.

Figure 5B shows the fits of the best-fitting Bayesian model (V5-CV). The model can capture the biases of Block ii across all groups even though it has less than half of the free parameters of the Linear model (6 vs. 13). The contrast functions for response and orientation bandwidth for all models can be seen in Supplementary Figures S2A and S2B, respectively. In the case of model V5-CV, both semi-saturation (8.17% and 8.96%) and exponent (2.35 and 2.31) values are very similar, suggesting that both response gain and orientation bandwidth barely change above 20% contrast and drop fastest at around 8% contrast. On average, models assuming contrast variance performed better than contrast invariant models suggesting that contrast variance in orientation bandwidth is required to explain the experimental results with the Bayesian model.

## Discussion

In the present study, we manipulated the orientation and contrast level of serially presented Gabor patches and measured the shift in their perceived orientation over time. We found that higher-contrast adaptors produce stronger serial effects than lower-contrast adaptors. More interestingly, we found a discrepancy in how the magnitude of serial effects on perception depend on the contrast of a stimulus and the sign of the effects. Repulsive aftereffects are stronger on the judgment of high rather than low contrast stimuli, whereas attractive aftereffects are stronger on the judgment of low-contrast stimuli. We described this discrepancy with a “linear” computational model where we disentangled serial effects of different signs and timescales, and we measured their magnitude on stimuli of different contrast levels. Then, we presented a Bayesian model observer where the two distinct types of aftereffects (attractive and repulsive) are generated from two distinct mechanisms that can explain the contrast-dependent magnitude of the aftereffects.

There is a clear correspondence between the three components of the linear model and the two mechanisms of the Bayesian model. The repulsive serial dependence with the immediate past (component 1) is explained by gain reduction in a network of orientation-tuned channels by the presentation of a stimulus. The medium-term repulsive aftereffect (component 2) is explained by the slow recovery of the gain over time. So, the repulsive effect of a stimulus is initially strong but decays over time. As low contrast stimuli elicit weaker responses on the network, the repulsive bias is smaller and so is the shift in their orientation likelihood. On the other hand, the long-term attractive aftereffect (component 3) is explained by the prior expectation developed over the course of the experimental session. As the rate of prior update is low, the attractive effect of a single stimulus is very weak, similar to the magnitude of component 3 in the Linear model. This is not contradictory with studies of attractive serial dependence because while the biases described in those studies are, on average, clear (e.g., Fischer & Whitney, 2014), the trial-to-trial variability is very high, suggesting an effect that is consistent but weak. Moreover, our Bayesian observer does not require a perfect memory of past distributions as is the case in some Bayesian models. It integrates each new stimulus sequentially, slowly updating an initially uniform prior. It is possible that a biased initial prior, for example toward cardinal directions, would be a more accurate prior but this is something that would require further assumptions and it is not necessary to explain our findings.

Our findings disagree with the prediction by Wei and Stocker (2015) that increasing sensory noise (i.e., reducing stimulus contrast) increases the repulsive bias but agree with the prediction for increasing stimulus noise (i.e., adding Gaussian noise), where repulsive biases change to attractive biases with increasing noise levels. A possible explanation for this is that decreasing contrast is not equivalent to reducing stimulus duration. Indeed, our model predicts that tuning widths change dramatically with decreasing contrast especially for levels under 10%. Furthermore, it predicts that changing stimulus duration is not equivalent to changing stimulus contrast. Supplementary Figure S3 (purple lines) shows the predictions of the model for each group of participants when presented with stimuli of high (50%) contrast but decreased duration equivalent to reduced contrast according to the parameters of the model (i.e., 10% contrast corresponds to a duration of 141.8 ms and 8% to 93 ms). These stimuli have the same exact likelihood in the absence of any serial effects. The simulations show that these stimuli are strongly affected by repulsive effects similar to the high contrast stimuli of regular duration (300 ms) for Groups B and C during Block ii. However, responses are almost identical for participants of Group C and during other Blocks.

The Bayesian model observer explains simultaneous attractive and repulsive aftereffects in a timescale larger than the model proposed by Fritsche, Spaak, and De Lange (2020), and it also proposes a specific implementation of the neural mechanisms that might be responsible for these aftereffects. The proposed mechanism for the repulsive aftereffects is similar to the gain reduction unit of the two-process model proposed by Pascucci et al. (2019). Our model reinforces and extends this idea by directly correlating the channel response inhibition to the channel activity induced by a stimulus and by showing that it depends on the stimulus contrast and duration. Moreover, it proposes a stimulus-independent recovery process that explains longer term repulsive traces. Regarding the attractive components of our and Pascucci et al.’s models, we argue that our prior information integration relates to much longer timescales than attractive serial dependence, and it is highly unlikely that it represents decision inertia. We have shown in the past that simply changing the decisional aspect of our task can reveal or hide strong attractive serial dependence (see Experiments 1 and 2 of Gekas et al., 2019). Finally, our Bayesian observer does not have to be optimal, even though it uses trial-to-trial variability uncertainty information (Ma, 2012).

However, several open questions remain. The model assumes that orientation-tuned channels very quickly reduce their response gain after stimulus presentation (see Ringach, Hawken, & Shapley, 1997). Mathematically, gain reduction is equivalent to a change in the orientation width of the tuning curve of the channel. So, it is possible that repulsive effects are caused by a change in the gain, a change in the width of the tuning curve, or a combination of the two. A more complex architecture of an orientation-tuned network is also possible, such as the one proposed by Gutierrez and Deneve (2019) where orientation is encoded by a network of high-gain and low-gain neurons. After sustained stimulus presentation, strongly excitable neurons are suppressed while weakly excitable neurons, especially those neighboring the adapting stimulus, increase their activity driven from the lack of inhibition from the high gain neurons. Such an architecture is difficult to test experimentally as a significant portion of neurons would need to be recorded locally before and after adaptation. Our experimental paradigm offers a potential way to test this hypothesis psychophysically. By systematically varying the contrast and the duration of the stimulus, it is possible to more accurately estimate the parameter ω (the accumulation of network activity over time) but also to measure the potential recruitment of low gain neurons. If indeed there is a tradeoff between efficiency and accuracy as Gutierrez and Denève suggest, even after prolonged exposure to an adaptor, we should not expect a large increase in aftereffects if low gain neurons are recruited to maintain accuracy. If, on the other hand, aftereffects increase proportionally with increased stimulus duration, a more complex network architecture seems less likely.

The visual system is extremely precise in orientation discrimination despite neurophysiological evidence suggesting that cortical visual neurons have broad orientation tuning (Vanni et al., 2020). Many studies have measured orientation discrimination as a function of contrast (Regan & Beverley, 1985; Skottun, Bradley, Sclar, Ohzawa, & Freeman, 1987; Reisbeck & Gegenfurtner, 1998), and these studies show that increasing contrast lowers orientation thresholds, albeit with a dependency on stimulus size (Mareschal & Shapley, 2004). While physiology studies in non-primate mammals have shown contrast-invariance in the tuning widths of V1 neurons (Finn, Priebe, & Ferster, 2007; Alitto & Usrey, 2004), Nowak and Barone (2009) argued that prolonged presentation of stimuli (two or four seconds) in these studies allowed the system to adapt to the stimulus contrast. So, instead, they tested primates (common marmoset) with brief presentations (200 ms) of grating stimuli. They found that orientation tuning in V1 neurons did not appear to be contrast-invariant for stimuli with variable orientations and contrasts. In our experiment, we used similar durations for our stimuli (300 ms) and a very large number of different orientations at two contrast levels. The comparison of different Bayesian models showed that, first, the response saturation profile of orientation-tuned channels better matched the profile of V5/MT neurons of the primate Macaca (Vanni et al., 2020), and second, that contrast-variance was necessary to describe the biases in participants’ responses with a saturation profile very similar to that of response gain. Although these findings should not be overstated, they offer a glimpse into the underlying neural properties of orientation perception and show how purely psychophysical studies can complement electrophysiological recordings.

## Supplementary Material

Supplement 1

Supplement 2

Supplement 3

Supplement 4

Supplement 5
