# Supplementary material for: Attractive and repulsive visual aftereffects depend on stimulus contrast
Source: J Vis. 2025 Jan 9;25(1):10. doi: 10.1167/jov.25.1.10 (PMC11725992; doi:10.1167/jov.25.1.10)
Supplement: Supplement 1 [file jovi-25-1-10_s001.pdf]

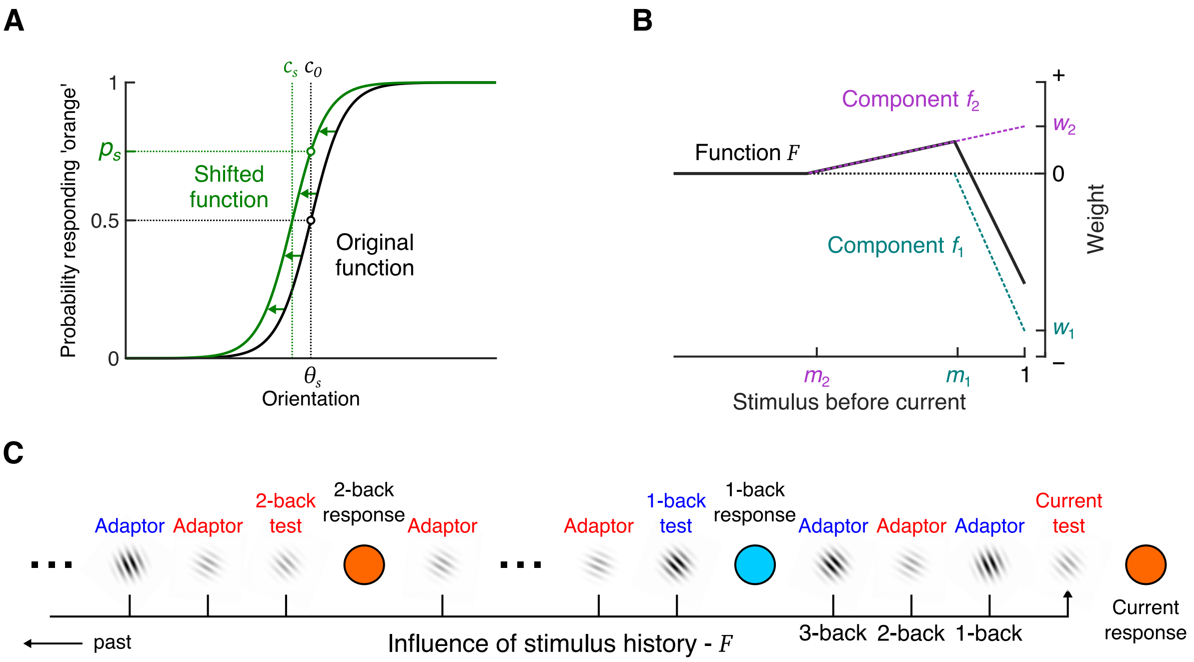


***Figure S1***. ***A.*** *The probability of responding 'orange' for a test stimulus $s$ with physical orientation $\theta_s$ is calculated from the psychometric function. When the midpoint of the function $c_s$ is shifted from the original midpoint $c_0$ by the history of past stimuli, a new probability of responding $p_s$ is calculated from the shifted psychometric function.* ***B.*** *Each component $f$ of the influence function $F$ is a linear function determined by the initial weight $w$ with the 1-back stimulus and the number of stimuli $m$ until the weight reaches zero. The influence function can be composed of an increasing number of components that sum up to a piecewise linear function.* ***C.*** *The perceived orientation of the current test is affected by the perceived orientations of past stimuli (adaptors and tests) according to function $F$.*
