# Supplementary material for: Attractive and repulsive visual aftereffects depend on stimulus contrast
Source: J Vis. 2025 Jan 9;25(1):10. doi: 10.1167/jov.25.1.10 (PMC11725992; doi:10.1167/jov.25.1.10)
Supplement: Supplement 2 [file jovi-25-1-10_s002.pdf]

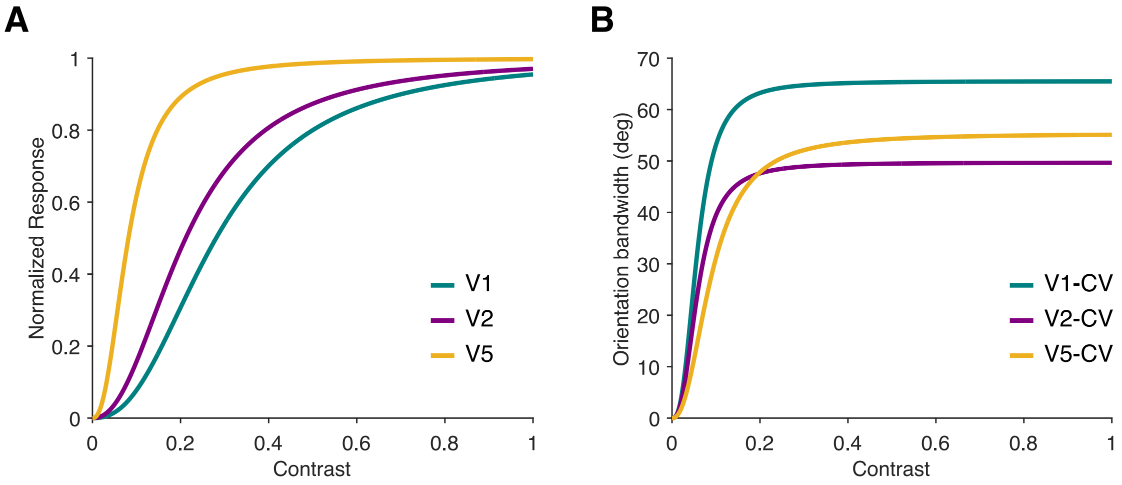

***Figure S2**. **A**. Contrast response functions, based on the median parameters reported in Vanni et al. (2020). **B**. Contrast orientation bandwidth functions, based on the parameters fitted by each Bayesian CV model reported in Table S1.*
