# Supplementary material for: Attractive and repulsive visual aftereffects depend on stimulus contrast
Source: J Vis. 2025 Jan 9;25(1):10. doi: 10.1167/jov.25.1.10 (PMC11725992; doi:10.1167/jov.25.1.10)
Supplement: Supplement 3 [file jovi-25-1-10_s003.pdf]

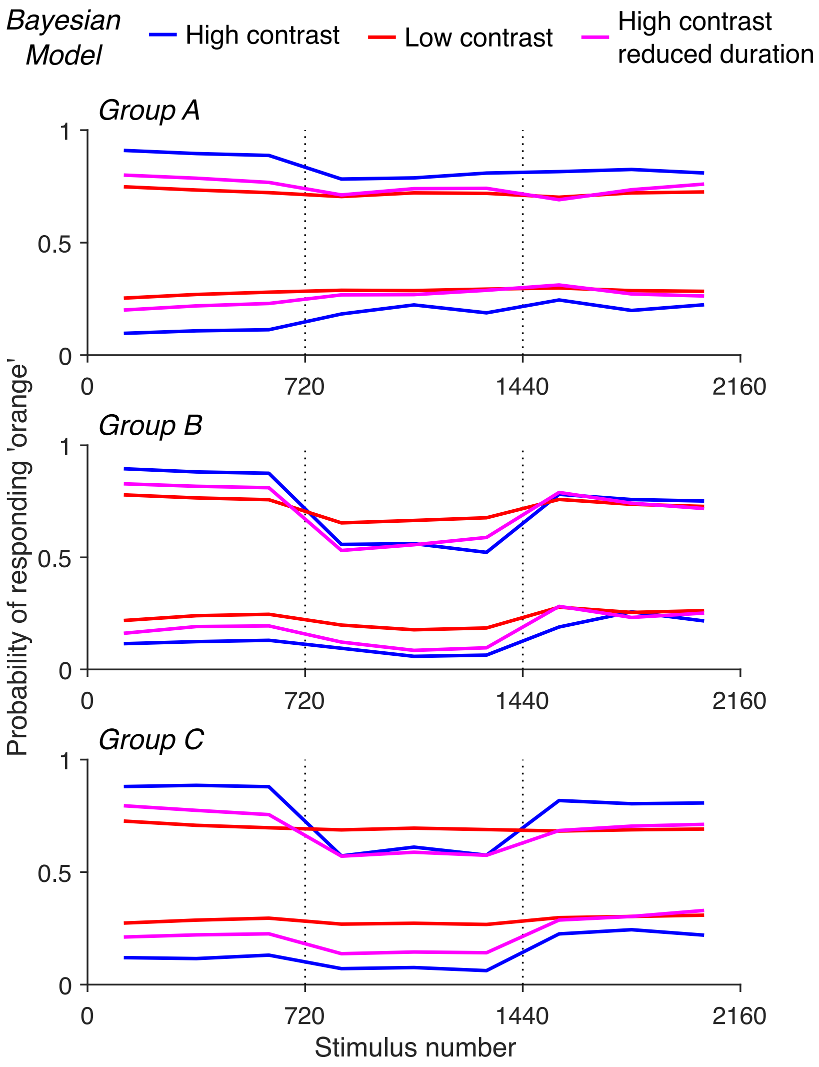

Figure S3. *The averaged probability of responding 'orange' to test stimuli as predicted by the best fitting Bayesian model (V5-CV) is plotted for each group of participants. Blue lines correspond to high contrast tests, red lines to low contrast tests, and purple lines to high contrast stimuli that are shown for reduced duration so that their unbiased likelihoods match those of the low contrast tests.*
