# Supplementary material for: Attractive and repulsive visual aftereffects depend on stimulus contrast
Source: J Vis. 2025 Jan 9;25(1):10. doi: 10.1167/jov.25.1.10 (PMC11725992; doi:10.1167/jov.25.1.10)
Supplement: Supplement 4 [file jovi-25-1-10_s004.pdf]

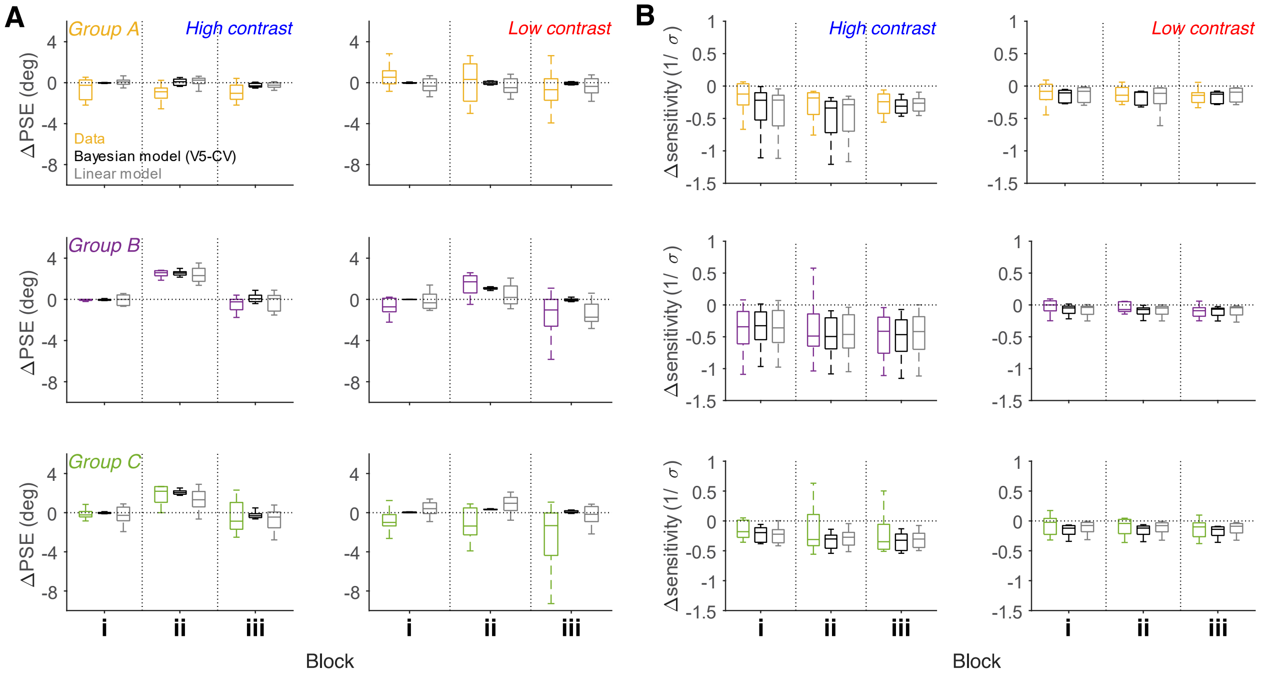

Figure S4. **A**. Box plots of the shifts in the PSE in comparison to the ones measured in Phase 1 are shown for each group (A at the top, B in the middle, and C at the bottom) for each block and for each contrast level (high contrast on the left, and low contrast on the right). Colored plots show the experimental data, black plots show the fits of the Bayesian model (V5-CV), and grey plots show the fits of the Linear model. **B**. Box plots of the shifts in sensitivity in comparison to the one measured in Phase 1.
