# Supplementary material for: Attractive and repulsive visual aftereffects depend on stimulus contrast
Source: J Vis. 2025 Jan 9;25(1):10. doi: 10.1167/jov.25.1.10 (PMC11725992; doi:10.1167/jov.25.1.10)
Supplement: Supplement 5 [file jovi-25-1-10_s005.pdf]

Table S1. Parameters of the Linear model.

Component	Initial weight (*w*)	Number of Stimuli (*m*)	Relative strength of high and low adaptors (*γ*)
High contrast f_1	-0.093	2	2.56
Low contrast f_1	-0.059	2	
High contrast f_2	-0.025	34	
Low contrast f_2	-0.003	89	
High contrast f_3	2.56 x 10^{-4}	2136	
Low contrast f_3	4.88 x 10^{-4}	2005	

Table S2. Parameters of the Bayesian models. Bolded parameters were allowed to vary to match the experimental data.

Symbol	Description	V1-CI	V1-CV	V2-CI	V2-CV	V5-CI	V5-CV
$\boldsymbol{\alpha}$	Gain reduction over network activity	0.011	0.0083	0.0222	0.0157	0.0403	0.0314
$\boldsymbol{\beta}$	Rate of maximum response recovery	0.3093	0.2763	0.2497	0.2839	0.3237	0.1915
$\boldsymbol{\tau}$	Rate of prior integration	4.79 x 10^{-6}	1.98 x 10^{-5}	8.08 x 10^{-5}	4.65 x 10^{-5}	2.55 x 10^{-4}	1.82 x 10^{-4}
$\boldsymbol{\omega}$	Accumulation of network activity over stimulus duration	57.91	60.90	21.78	22.77	11.64	15.53
$\boldsymbol{w_{50}}$	Contrast semi-saturation over tuning width	NA	0.0581	NA	0.0591	NA	0.0896
$\boldsymbol{\varepsilon}$	Contrast exponent over tuning width	NA	2.6845	NA	2.5612	NA	2.3096
c_{50}	Contrast semi-saturation over maximum response	0.2807	0.2807	0.2116	0.2116	0.0817	0.0817
c_{exp}	Contrast exponent over maximum response	2.401	2.401	2.245	2.245	2.351	2.351
κ_{φ}	Channel maximum spread	65.52	65.52	49.69	49.69	55.31	55.31
